# Chemical composition, characterization, and antimicrobial properties of *Thymbra spicata* essential oil-based nanoemulsions and its application on curd cheese

**DOI:** 10.3389/fnut.2025.1615832

**Published:** 2025-06-18

**Authors:** Serpil Demirci Kayiran, Umay Merve Guven Bolgen, Ebru Akgül, Pinar Kadiroglu, Nurten Cengiz, Tilbe Cevikelli, Deniz Onan, Esra Eroglu Ozkan, Gizem Bozdogan, Mehmet Boga, Yesim Ozogul, Tuba Esatbeyoglu, Fatih Ozogul

**Affiliations:** ^1^Department of Pharmaceutical Botany, Faculty of Pharmacy, Cukurova University, Adana, Türkiye; ^2^Department of Pharmaceutical Technology, Faculty of Pharmacy, Cukurova University, Adana, Türkiye; ^3^Department of Food Engineering, Faculty of Engineering, Adana Alparslan Turkes Science and Technology University, Adana, Türkiye; ^4^Department of Pharmaceutical Technology, Faculty of Pharmacy, Bahçeşehir University, Istanbul, Türkiye; ^5^Department of Pharmaceutical Biotechnology, Faculty of Pharmacy, Cukurova University, Adana, Türkiye; ^6^Department of Pharmacognosy, Faculty of Pharmacy, Istanbul University, Istanbul, Türkiye; ^7^Department of Analytical Chemistry, Faculty of Pharmacy, Dicle University, Diyarbakir, Türkiye; ^8^Department of Seafood Processing Technology, Faculty of Fisheries, Cukurova University, Adana, Türkiye; ^9^Department of Molecular Food Chemistry and Food Development, Institute of Food and One Health, Gottfried Wilhelm Leibniz University Hannover, Hannover, Germany; ^10^Biotechnology Research and Application Center, Cukurova University, Adana, Türkiye

**Keywords:** *Thymbra spicata*, essential oil, nanoemulsion, antimicrobial activity, food preservation, curd cheese, antioxidant properties

## Abstract

**Background and aims:**

*Thymbra spicata* L. has been widely recognized as a food additive due to its therapeutic benefits and low toxicity risk. This study focuses on the chemical constituents of *T. spicata* essential oil, antimicrobial, and antioxidant properties of essential oil (EO)-based nanoemulsions (NEs) and for the first time their effect on the preservation of curd cheese.

**Methods:**

Nanoemulsions containing three different concentrations of EO (1%, 3%, and 5%) were tested for conductivity, droplet size, pH, polydispersity index (PDI), rheological properties, viscosity, and zeta potential. In addition, chemical composition was analyzed using gas chromatography-mass spectrometry. Moreover, the antimicrobial properties of the EOs and its nanoemulsion forms were evaluated *in vitro* against *Staphylococcus aureus, Escherichia coli, Pseudomonas aeruginosa*, and *Bacillus subtilis* using the agar well diffusion assay. After *in vitro* studies, the nanoemulsion concentrations of 1%, 3%, and 5% and pure EO were incorporated into curd cheese. Curd cheese treatments were assessed in terms of total yeast and mold, mesophilic aerobic and lactic acid bacteria count, and quality analyses for 28 days.

**Results:**

The results showed that the dispersions with a spherical shape and droplet sizes were smaller than 165 nm for all three concentrations (154.9, 165.0, and 151.3 nm, respectively). All formulations maintained their physical properties even after stability tests. Major components in the essential oil (EO) were identified as γ-terpinene, thymol, carvacrol, and p-cymene. The nanoemulsion delayed the growth of mold and yeast for up to 28 days.

**Conclusions:**

Consequently, these findings indicate that *T. spicata* EO and derived nanoemulsions could be used as vital sources for developing new and impactful antimicrobial agents for food and different industries.

## 1 Introduction

Food safety is the main concern of producers, regulators, and consumers. Foodborne pathogens pose a public health risk, and despite current breakthroughs in production procedures, the frequency of foodborne infections is not reducing effectively (1, 2). Essential oils (EOs) have gained widespread commercial use across various industries due to their aromatic, therapeutic, and antimicrobial properties. For instance, lavender EO is frequently incorporated into skincare products such as Aveeno Stress Relief Moisturizing Lotion for its calming effects, while peppermint oil is found in Dr. Bronner's Peppermint Pure-Castile Soap and Burt's Bees Cooling Lip Balm for its refreshing and soothing properties. Tea tree oil, known for its antimicrobial activity, is a key ingredient in The Body Shop Tea Tree Oil line, targeting acne, and skin blemishes. Additionally, eucalyptus oil is used in Vicks VapoRub for its respiratory benefits (3). Growing consumer demand for foods with fewer chemical additives has boosted interest in essential oils (EOs). Rich in terpenoids and phenolic acids, EOs exhibit potent antibacterial, antifungal, and antioxidant activities (1). These natural, eco-friendly compounds deliver broad-spectrum microbial suppression by allowing the hydroxyl groups on their phenolic rings to interact with and disrupt microbial cell membranes, making them an effective alternative for extending the shelf life of various food products (2).

Currently, the food industry has shown an increasing need for formulations based on natural products to create new food preservatives that can stop the growth of microorganisms, extend the shelf life of food, and preserve food in different storage conditions (4). EOs are natural, volatile compounds extracted from plants. They are widely utilized in numerous sectors, particularly in the food and pharmaceutical industries. In the pharmaceutical sector, EOs are used in medication topical formulations, aromatherapy, and alternative medicine owing to their antibacterial, anti-inflammatory, antioxidant, and therapeutic properties (5). EOs on the “Generally Recognized As Safe” (GRAS) list have emerged in recent years as attractive antimicrobial agents with significant potential for use in bioactive food packaging. However, even GRAS-status EOs face limitations regarding their permissible concentrations and the types of foods to which they can be applied due to factors such as potential sensory impacts and regulatory restrictions. Plenty of research has investigated the bioactivity of EO-based nanoemulsions (NEs) to address these challenges and enhance their effectiveness and applicability (5–7). There are some studies of EO-based nanoemulsion on cheese (8, 9). Cai et al. (10) reported that the orange EO-based nanoemulsion with guar gum/chitosan edible films was effective for the preservation of cheese. Faramarzi et al. (11) investigated the inhibitory effect of *Urtica dioica* EO-based nanoemulsion and placebos on the food pathogen bacteria in pizza cheese. Rossi et al. (12) assessed the antimicrobial activity of Cinnamon EO-based nanoemulsions against *Pseudomonas paracarnis* in fresh cheese.

Nanoemulsions (NEs) are heterogeneous dispersions of colloidal particles composed of two separate liquid phases (oil and water phases) stabilized by surfactants and cosurfactants. NEs are optically transparent with a mean droplet diameter of <200 nm. This transparency is crucial in their widespread use in developing clear food products and drug delivery systems (13, 14). NEs are prepared using low-energy methods by exploiting the chemical capacity of their components and high-energy methods based on shearing and homogenization under high pressure. Low-energy approaches are preferred for thermosensitive compounds since they impose lower energy demand. Spontaneous emulsification enhances the production of nanometric droplets by increasing the surface area of the oil–water interface, compared with the use of fixed carrier oils for stability due to a significant proportion of medium-chain fatty acids (15). Nanoemulsions are especially useful for EOs due to their ability to enhance stability, protect volatile components from environmental degradation, and improve water dispersibility. Their small droplet size increases surface area, enabling better bioavailability and antimicrobial activity. Additionally, nanoemulsions allow for controlled and sustained release, which prolongs the functional effects of EOs in various applications (5, 13).

Bioactive compounds, such as certain polyphenols, carotenoids, and EOs, which have low solubility in polar solutions such as water, are less soluble in polar solutions, which may limit their dispersion and interaction within aqueous food systems, potentially reducing their effectiveness in preserving food. NE formulations have a small droplet size (O/W system), which enhances the solubility of the components via surfactants and the absorption of the formulation. Many previous studies have used NEs as carrier systems for evaluating the activity of various EOs, such as *Foeniculum vulgare, Ocimum, Tribute citrus*, cinnamon, and thyme. *Thymbra spicata* is an aromatic plant that belongs to the Lamiaceae family. Previous studies on EO nanoemulsions have demonstrated that formulating EOs into nano-sized droplets significantly enhances their stability, solubility, and bioavailability, especially in aqueous environments. These studies also highlighted improved antimicrobial and antioxidant properties due to the increased surface area and better dispersion. Inspired by these findings, we aimed to explore whether a similar nanoemulsion formulation could enhance the biological activity of *T. spicata* EO, which is known for its rich phenolic content and strong bioactivity but suffers from volatility and poor water solubility. The goal was to apply the benefits observed in earlier EO nanoemulsion work to a less-studied but highly promising plant species (16–20).

In Mediterranean countries, it is used as medicine or food, adding flavor to meat dishes, soups, and salads. *T. spicata* EO comprises many chemical compounds such as thymol, p-cymene, carvacrol, gamma-terpinene, and caryophyllene (21, 22). *T. spicata* EO is highly valuable in the pharmaceutical and cosmetics industries owing to its potent aroma and therapeutic properties. It has demonstrated antimicrobial, antioxidant, antifungal, anti-biofilm, cytotoxic activities, and anti-quorum-sensing (23–25).

Gedikoglu and Çikrikci Erünsal (26) reported that *T. spicata* EO-based NEs revealed an extremely high antibacterial activity against *S. epidermidis, B. cereus, E. coli*, and *Salmonella enteritidis*. Previously, *T. spicata* EOs were evaluated by Gedikoglu (27) for their antimicrobial activity against *S. typhimurium* and antioxidant activity in pectin-based edible coatings for aerobically packaged, ready-to-eat, sliced Bologna meat products during cold storage. Sengun et al. (28) examined the impact of *T. spicata* EO and its extract against *S. aureus*, revealing that the EOs more effectively inhibited the growth of *B. subtilis* and *L. monocytogenes*. However, no study has yet evaluated *T. spicata* EO-based nanoemulsions in preserving curd cheese under real storage conditions.

In this study, the chemical composition of the *T. spicata* EO was analyzed using GC-MS. Besides, *in vitro* and *in vivo* antimicrobial activities and antioxidant properties were tested using the EO-based nanoemulsions. There are a limited number of studies on the use of *T. spicata* EO-based nanoemulsions in cheese preservation. Therefore, this study focuses on the chemical constituents of *T. spicata* EO, antimicrobial and antioxidant properties of EO-based nanoemulsions, and their effect on the preservation of curd cheese.

## 2 Materials and methods

### 2.1 Plant material

*Thymbra spicata* plant samples used in this study were obtained from the herbal market in Adana, Southern Turkey, in August 2024. The samples were determined by S. Demirci Kayiran.

### 2.2 Preparation of *T. spicata* EO

The plant material was allowed to dry in the shade at room temperature. A Clevenger apparatus (ISOLAB) was used to hydrodistillate a 50-g portion of the dried materials with distilled water (250 ml) for 3 h. After three repetitions of the procedure, the resulting EO was stored for later usage at 4°C.

### 2.3 GC-MS analysis of *T. spicata* EO

The *T. spicata* EOs were analyzed using an Agilent MS system (7010B) coupled to an Agilent GC (7890B; Agilent Technologies Inc., CA, USA).

The EOs were analyzed using Gas Chromatography–Flame Ionization Detection/Mass Spectrometry (GC-FID/MS) instrument, following the protocol of Demirci Kayiran et al. (29). GC analyses were performed using a DB-Wax column (60 m × 0.25 I.D., film thickness 0.25 μm; J&W Scientific, CA, USA). The injector and detector temperatures were set to 250°C. Helium, with a flow rate of 1.4 ml/min, was the carrier gas. The split ratio was 20:1, and 1.0 μl was used as the sample size. The initial temperature of the oven was kept at 40°C for a period of 4 min, then elevated up to 250°C in steps of 5°C/min, and could be kept at this temperature for 10 min. The NIS14L software was used to calculate the percentage composition of the EO.

### 2.4 Construction of pseudo-ternary phase diagrams

The NE formulations were prepared by the pseudo-ternary phase diagram method. The oil phase, surfactant, cosurfactant, and aqueous phase were used while constructing the diagrams, which were oleic acid, Cremophor EL, ethanol, and distilled water, respectively. Combinations of surfactant and cosurfactant (S/CoS or *S*_mix_) were investigated with weight ratios of 1:1, 1.5:1, and 2:1. Nine different concentrations were used to achieve the oil-to- *S*_mix_ ratios of 1:9, 2:8, 3:7, 4:6, 5:5, 6:4, 7:3, 8:2, and 9:1. All the preparations were titrated with distilled water at room temperature with continuous mixing using a mechanical stirrer (HEIDOLPH; 600 rpm). Distilled water was added to the system until it began to cloud, and the volume was recorded. Computer software was utilized to create pseudo-ternary phase diagrams to determine the percentages of oil, *S*_mix_, and distilled water during NE formulation (30).

### 2.5 Preparation of NE formulations by low-energy methods

The formulation that yielded an NE system was chosen based on the phase diagram results. The best NE ratio was determined to be at the center of the NE-forming regions. Selecting the NE ratio from the center of the NE-forming region is a rational approach, as it reflects optimal component balance and promotes thermodynamic stability. This region typically yields homogeneous, clear, and kinetically stable systems with smaller droplet sizes and improved physical stability. The formulation was prepared by titrating an oil and *S*_mix_ with distilled water at room temperature with mechanical stirring using a low-energy method, similar to the preparation of pseudo-ternary phase diagrams. *T. spicata* EO was used at various concentrations (1%, 3%, and 5%) by dissolving it in the oil phase. The placebo was achieved by preparing the formulation using the same process as *T. spicata* EO but without the oil (15, 31).

### 2.6 Characterization of NE formulations

#### 2.6.1 Droplet size, distribution, and zeta potential

The droplet size and the droplet size distribution of placebo and EO-based NEs were analyzed by dynamic light scattering (DLS) technique (Horiba SZ100, Japan). In this analysis, a 532-nm laser and scattering angles of 90 and 173° were used by the HORIBA SZ-100 DLS instrument, depending on sample conditions. Average droplet size was measured, and data were presented as average diameter ± standard deviation (SD). The zeta potential was determined by means of disposable flat-fold capillary zeta cells, and the measurements were performed through the system software. Measurements were repeated three times to obtain the mean millivolt ± SD at 25°C (32).

#### 2.6.2 Morphology of NEs

The microscopic image was used to analyze the shape of the microemulsions using a high-resolution inverted microscope (Leica DM IL LED Fluo, Germany). The results were represented in corresponding figures (33).

#### 2.6.3 pH value

The pH value of placebo and EO-based NE formulations was measured at 25 ± 1°C with a digital pH meter (Mettler Toledo FiveGo, Switzerland). The test was achieved in triplicate and displayed as mean ± SD (34).

#### 2.6.4 Conductivity

The electrical conductivity of placebo and EO-based NEs was determined using a conductivity meter (Mettler Toledo FiveEasy Plus). The samples were kept at 25 ± 1°C. The measurements were accomplished in triplicate and pointed out as mean ± SD (35).

#### 2.6.5. Viscosity and rheological properties

A Brookfield cone and plate rheometer were used to measure the shear stress, shear rate, and apparent viscosity of placebo and EO-based NEs in triplicate (Brookfield DV3THACJ0; Brookfield Engineering Labs. Inc., MA, USA) under controlled temperature (25 ± 0.5°C). Rotation speed was adjusted at 10–100 rpm (31).

### 2.7 Stability of NE formulations

Thermodynamic stability assays were conducted to elucidate the long-term stability of the formulations. The formulations were evaluated by centrifugation and cycles of freeze-thaw and heating-cooling. Each formulation was centrifuged using a centrifuge (5,000 rpm for 30 min, performed in triplicate). In addition, the formulations were subjected to a cycle of heating and cooling and then stored at 4 and 45°C, respectively, for 48 h. This was followed by a cycle of freeze-thaw. The formulations were placed in vials and frozen at −4°C for 12 h and at room temperature for 12 h. Each experiment was performed in triplicate (36, 37).

### 2.8 Antimicrobial activity analysis of EO and NE formulations

The agar well diffusion assay was applied to determine antimicrobial activities using *B. subtilis, P. aeruginosa, S. aureus, and*. *E. coli*, which were activated by incubation at 37°C for 24 h. After activation, the bacteria were mixed with sterile 0.9% NaCl solution and adjusted to a density of McFarland 0.5 (~10^8^ cfu/ml). This bacterial solution (100 μl) was applied to the Muller–Hinton agar (MHA) medium by spreading method. Then, 7 mm diameter wells were made on the medium, and 100 μl of the extract was added to each well. The concentrations of the extracts ranged from 100 to 0.315 mg/ml. Petri dishes were incubated at 37°C for 24–48 h. Following incubation, the diameters of the inhibition zones around the wells were measured with a caliper. The well diffusion test was performed in two parallel runs for each petri, and the diameter of the inhibition zones was measured at three different points (38).

### 2.9 Application of the nanoemulsions on the curd cheese

Curd cheese was obtained daily from the Dairy Products Processing Unit of the Food Branch under the Directorate of the Research and Application Farm of the Faculty of Agriculture, Çukurova University. Curd cheese was classified into six groups: control (plain curd cheese), *T. spicata* EO, nanoemulsions containing 1%, 3%, and 5% *T. spicata* EO, and placebo. Each group was prepared in 500-g portions. Curd cheese of ~100 g was put into single-use plastic containers and stored for 28 days at 4 ± 1°C in a refrigerator. Antioxidant activity, total phenolic content (TPC), pH, color parameters, and microbiological analysis of these curd cheese samples were determined at 0, 3, 5, 7, 14, 21, and 28 days of storage period. To minimize potential carry-over effects of residual EOs, the EO-containing agar media were allowed to stand under a laminar flow hood for a predetermined period before inoculation. This step was applied to allow the volatile compounds to stabilize before microbial analysis.

#### 2.9.1 Physicochemical analysis of cheese samples during storage

The cheese sample (10 g) was homogenized with 100 ml distilled water for 1 min using a homogenizer. The pH was carried out using a digital pH meter (Mettler-Toledo, Schwerzenbach, Switzerland), according to the method of Polat Yemiş et al. (39). Color measurements were carried out at room temperature to ensure consistency and minimize temperature-related variations using a Minolta Chroma meter (Chroma Meter CM-5; Minolta Camera Co. Ltd., Osaka, Japan), following the method of Cai et al. (10). The values of L^*^, a^*^, and b^*^ were done and the whiteness index (WI) was calculated according to the following equation (40).


(1) $$\begin{array}{l} WI\;=\;100\;-\;{\left[{\left(100\;-\;L^{*}\right)}^{2}+\;(a^{*2}+\;b^{*2})\right]}^{0.5} \end{array}$$

#### 2.9.2 Antioxidant activity and total phenol compounds

Antioxidant activity and total phenol compounds were accomplished by using the method of Shawir et al. (41). For antioxidant activity (ABTS) and total phenolic content, the samples, weighing 5 g, were placed completely in 10 ml of methanol and then centrifuged at 5,000 rpm for 10 min.

#### 2.9.3 ABTS assay

The ABTS method was done with slight modifications as described by Re et al. (42). The reaction between the diluted sample and 3.9 ml ABTS radical solution (7 mM; mixed with 2.45 mM potassium persulfate and left for 12–16 h) was carried out kinetically by measuring the change in 734 nm absorbance (Cary 60 UV-Vis Spectrophotometer, Agilent Technologies) for 15 min. Antioxidant activities were expressed as μmol Trolox/L from the absorbances of Trolox standards prepared at different concentrations (6.25–200.0 ppm) determined by the same method.

#### 2.9.4 Total phenolic content analysis

Total phenolic content (TPC) analysis was done according to the method of Singleton et al. (43) with minor changes. Before analysis, 0.5 ml of Folin–Ciocalteu solution was added to cheese samples prepared according to the procedure of Shawir et al. (41), and the mixture was left for 3 min. After that, 20% sodium carbonate was added to this mixture and allowed to stand for 60 min at room temperature in the dark room. Absorbance values were read at 765 nm with a UV/VIS spectrophotometer (Cary 60, Agilent Technologies). Using the calibration received from the absorbances of standards (gallic acid) prepared at different concentrations (between 31.25 and 500.0 ppm) by the same method, the TPC values were then calculated. Data were presented as mg/g of gallic acid equivalents.

#### 2.9.5 Microbiological analysis of cheese samples during storage

Lactic acid bacteria (LAB), total aerobic mesophilic bacteria (TAMB), and yeast–mold assays were performed on curd cheese samples. The results were calculated as log cfu/g.

##### 2.9.5.1 Determination of total aerobic mesophilic bacteria counts (TAMB)

TAMB counts in curd cheese samples were determined by inoculation on plate count agar medium using the pour plate method. Following inoculation, colonies that developed after 48–72 h of incubation at 30°C were counted (44, 45).

##### 2.9.5.2 Determination of yeast and mold counts

Yeast and mold counts were determined using the spread plate counting method on Yeast Extract Glucose Chloramphenicol Agar medium. Following inoculation, yeast colonies were counted on the third day, and mold colonies were counted on the fifth day of incubation at 30°C (44).

##### 2.9.5.3 Determination of lactic acid bacteria count

Enumeration of lactic acid bacteria was performed using the pour plate method, administering De Man, Rogosa, and Sharpe Agar (MRS). The sample (10 g) was blended with 10 ml of distilled water, serially diluted, and 1 ml of the appropriate dilution was transferred into sterile petri dishes. Then, molten MRS agar was poured over the sample, gently mixed, and allowed to solidify. The plates were incubated at 30°C for 3 days. After incubation, LAB colonies were counted and expressed as log cfu/g (46). All experiments were carried out in triplicate.

### 2.10 Statistical analysis

Data analysis of ANOVA Fisher's Least Significant Difference (level of confidence 95%, *p* < 0.05) was performed using Minitab^®^ 17 software (Minitab Inc., State College, PA, USA). The values are expressed as the mean ± standard deviation. Statistical analysis was performed using one-way ANOVA followed by Tukey's *post-hoc* test to compare the physicochemical properties of different nanoemulsion formulations (NE 2:1 Placebo, 1%, 3%, and 5%). A *p*-value of < 0.05 was considered statistically significant. The analysis showed no significant differences in pH and conductivity among the formulations (*p* > 0.05), while droplet size and viscosity exhibited minor but non-significant variations. Notably, the polydispersity index (PDI) of the 1% formulation was significantly higher than that of the placebo (*p* < 0.05), indicating a broader size distribution at this concentration.

## 3 Results and discussion

### 3.1 Chemical components of EOs

The analysis of *T. spicata* EO revealed that its chemical composition is dominated by four major compounds, including γ-terpinene (25.54%), thymol (22.47%), carvacrol (19.26%), and *p*-cymene (17.74%; Table 1). These constituents accounted for ~85% of the total oil content, underscoring their primary role in the biological activities of the oil. The predominance of γ-terpinene was consistent with previous findings on *Thymbra* and *Thymus* species (47). As a monoterpene hydrocarbon, γ-terpinene serves as a biosynthetic precursor for oxygenated monoterpenes such as carvacrol and thymol (48). The high levels of thymol and carvacrol in this study aligned with the thymol-carvacrol chemotype widely reported in *T. spicata* populations (49).

**Table 1:** Chemical composition of *Thymbra spicata* EO.

**No**	**Constituents**	**Retention time (minutes)**	**Relative peak area (%)**	**Retention index**
1	1R-α-Pinene	11.05	1.03	1,011
2	β-Thujene	11.25	1.67	1,133
3	β-Myrcene	17.56	2.04	1,160
4	α-Terpinene	18.38	3.67	1,176
5	γ-Terpinene	21.77	25.54	1,196
6	β-Ocimene	21.93	0.66	1,240
7	p-Cymene	22.73	17.74	1,252
8	Linalool	34.30	1.01	1,266
9	Terpinen-4-ol	37.10	0.9	1,549
10	Caryophyllene	37.74	3.03	1,601
11	Caryophyllene oxide	50.01	0.66	1,612
12	Thymol	53.57	22.47	2,005
13	Carvacrol	54.24	19.26	2,185
	Total		99.68	

The ratios of thymol and carvacrol vary based on environmental factors such as geographical location, climate, and extraction methods. Studies on *T. spicata* species collected from various regions of Turkey have revealed variations in the chemical composition of their EOs. In a previous study, the EOs extracted from *T. spicata* leaves collected from Nizip and Gaziantep using subcritical water extraction were found to be mainly composed of carvacrol (86.2%), thymol (3.67%), and E-3-caren-2-ol (3.08%) (50), whereas the EO obtained through hydrodistillation of *T. spicata* collected from Diyarbakir was predominantly composed of thymol (55.3%), carvacrol (8.7%), caryophyllene (4.2%), and p-cymene (11.2%) (47). Inan et al. (51) found that the highest proportion (64.53%) of carvacrol was discovered in the EO of *T. spicata* collected from Kahta, Adiyaman, after flowering, whereas the lowest proportion (53.55%) was observed before the flowering stage. Moreover, thymol was not detected in the EO. Another study determined the EO content of *T. spicata* var. spicata collected from Aksu, Antalya. The most abundant compounds were carvacrol (63.23%), *p*-cymene (8.31%), and γ-terpinene (18.94%) (52). Additionally, the composition of the EO obtained by hydrodistillation from *T. spicata* collected from Eskişehir revealed that the major compounds were carvacrol (56.03%), *p*-cymene (9.61%), γ-terpinene (6.87%), and *trans*-caryophyllene (10.41%) (28). Moreover, Nath et al. (53) studied the chemical composition of the EO of *T. spicata* collected from Kepsut, Balikesir, and the results revealed that the main components of *T. spicata* EO were carvacrol (52.3%) and *p*-cymene (21.1%).

Studies indicate that the oils from warmer regions have higher carvacrol content, whereas those from cooler climates are more likely to produce thymol (54). The composition reported in the present study suggests a balanced synthesis of these phenolic compounds, reflecting the influence of local environmental conditions on the EO profile.

From a biological perspective, thymol and carvacrol are well-documented for their potent antimicrobial, antioxidant, and anti-inflammatory activities (55). Their presence at elevated levels suggests significant therapeutic potential of the *T. spicata* EO. In addition, γ-terpinene and *p*-cymene exhibit synergistic effects with thymol and carvacrol, enhancing their biological efficacy (56). This synergism may contribute to the enhanced antimicrobial properties commonly associated with *T. spicata* EO (57).

### 3.2 Pseudo-ternary phase diagrams and preparation of NE formulations

The pseudo-ternary phase diagram method was used to determine the NE formation regions and obtain a clear and stable NE. Thus, a pseudo-ternary phase diagram was used to obtain the best concentration ratios of surfactant, co-surfactant (*S*_mix_), and oil. Different concentration ratios ranging from 1:9 to 9:1 of Cremophor EL and ethanol were used as the surfactant-co-surfactant mixtures to prepare *S*_mix_. Cremophor EL is well known for its outstanding emulsifying activity in the development of NEs. These surfactants are non-toxic; hence, they create oil-in-water NEs with high stability (58–60). Akhter et al. (61) successfully prepared an NE gel using an extract of *Cucumis melo*. Ethanol was chosen as the cosurfactant in the formulation since short-chain alcohols are commonly used as cosurfactants to enhance NE formation. The NE regions of the diagrams obtained with three different *S*_mix_ ratios were determined. The compositions corresponding to the central points of NE regions are shown in Table 2.

**Table 2:** Blank nanoemulsion compositional ratios and relative nanoemulsion areas from pseudo-ternary phase diagrams.

**Formulation (% weight)**			
**Components**	**NE1:1**	**NE1.5:1**	**NE2:1**
Oleic acid	15.37	15.74	16.25
Cremophor^^®^^ EL	28.25	35.59	39.88
Ethanol	28.25	23.73	19.94
Water	28.13	24.94	28.93
NE area	427.79	402.79	438.52

NE, nanoemulsion; NE1:1, the rates of 1%; NE1.5:1, the rates of 3%; NE2:1, the rates of 5%.

**Figure 1:**
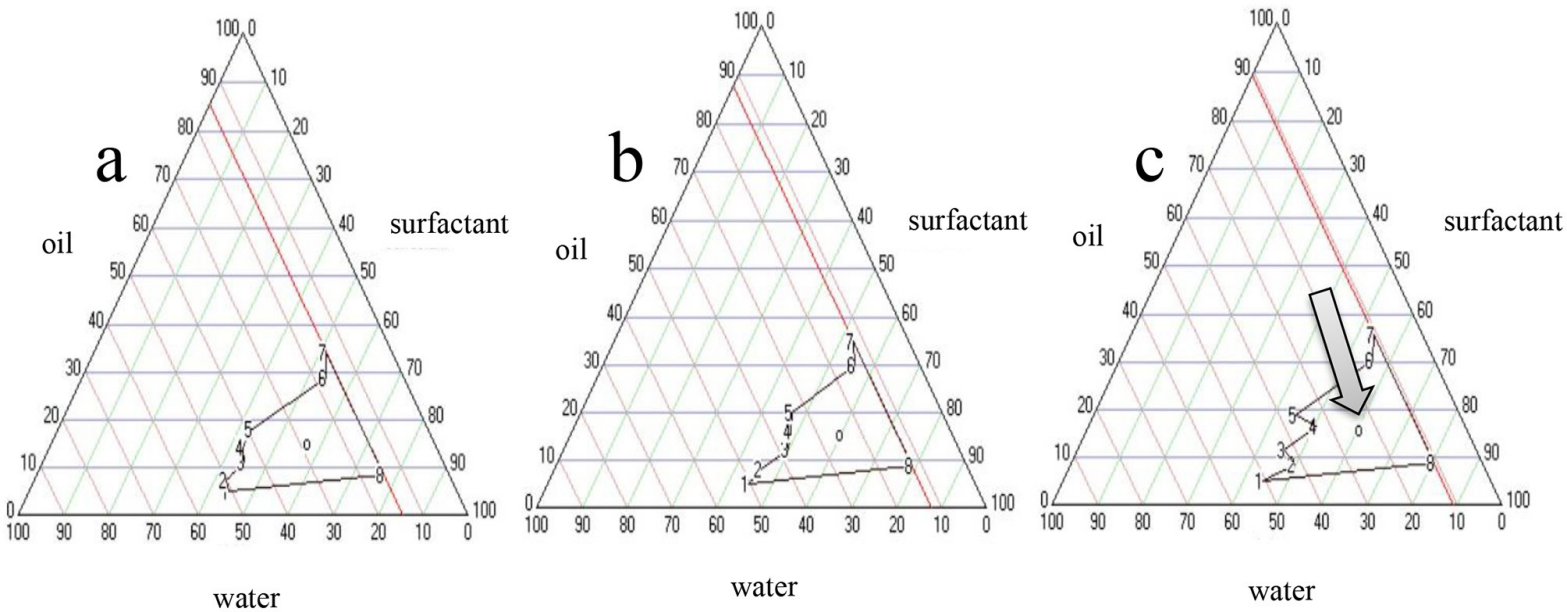
Pseudo-ternary phase diagrams of nanoemulsions. **(a)** NE1:1, **(b)** NE1.5:1, **(c)** NE2:1.

The NE formation region in the pseudo-ternary phase diagram is shown in Figure 1. Notably, a distinct order of NE formation region was seen where the ratio of *S*_mix_ followed the order *S*_mix_ 2:1 > *S*_mix_ 1:1 > *S*_mix_ 1.5:1. The NE formation had a larger region with a *S*_mix_ ratio of 2:1, indicating that NE formation was optimal at a *S*_mix_ ratio of 2:1. Therefore, this formulation was chosen for characterization and activity tests.

Turbidity or transparency of NE formulations not only provides a good appearance but also serves as a crucial parameter indicating thermodynamic stability. Transparent, homogeneous, and non-turbid NEs are more stable than conventional emulsions, with higher shelf life (61, 62).

### 3.3 Characterization of nanoemulsions

The oil phase was prepared by adding *T. spicata* EO at the rates of 1%, 3%, and 5% (*w*/*w*) concentrations. The optimal NEs displayed a yellowish color with translucency against a blue background and carried the distinctive aroma of *T. spicata* EO. The characteristics of the formulation were evaluated in terms of droplet size and distribution, pH value, zeta potential, viscosity, and rheological properties.

#### 3.3.1 Droplet size, distribution, and zeta potential

One of the most essential properties for stability is droplet size and distribution. The concentration of oil and *S*_mix_ has a synergy with the droplet size. Besides the small droplet size, the distribution is also important and is quantified using the polydispersity index (PDI) (26, 62). Wei et al. (63) reported that the droplet size of fenpropathrin NE decreased by increasing the concentrations of surfactant and cosurfactant, and the stability of the emulsion also improved. Similar results were found by Salama et al. (64).

The PDI is a parameter that measures the homogeneity of droplets, ranging between 0.0 and 1.0. A low PDI value indicates closely packed droplet size and homogeneity in the distribution of NE formulations. A PDI value ≤ 0.7 corresponds to a monodisperse formulation, suggesting a uniform NE. A PDI value < 0.25 signifies a narrow size distribution in the system. The other values indicate that the formulations are in acceptable ranges (30, 65). The average droplet size and PDI values of optimum formulations are shown in Table 3.

**Table 3:** Mean droplet size, PDI, zeta potential, pH, conductivity, and viscosity obtained for the characterization of nanoemulsions [mean ± standard deviation (SD), triplicate].

**Formulations code**	**Droplet size (nm ±SD)n**	**PDI**	**Zeta potential (mV ±SD)**	**pH**	**Conductivity (μSm/cm ±SD)**	**Viscosity (mPa·S ±SD)**
NE2:1 Placebo	150.6 ± 21.1	0.359 ± 0.070	0.01 ± 0.03	5.47 ± 0.03	0.196 ± 0.02	126 ± 3
NE2:1 % 1	154.9 ± 9.6	0.620 ± 0.131	0.00 ± 0.00	5.47 ± 0.02	0.202 ± 0.04	118 ± 13
NE2:1 % 3	165.0 ± 3.6	0.492 ± 0.146	0.02 ± 0.03	5.49 ± 0.02	0.196 ± 0.02	110 ± 9
NE2:1 % 5	151.3 ± 15.6	0.495 ± 0.219	0.01 ± 0.00	5.51 ± 0.02	0.201 ± 0.02	119 ± 17

n, three measurements; PDI, the polydispersity index; mPa·s (millipascal-second), mV ± SD, measured in millivolts (mV), expressed as mean ± standard deviation (SD). μSm/cm ± SD, Electrical conductivity (μS/cm), expressed as mean ± standard deviation (SD).

**Figure 2:**
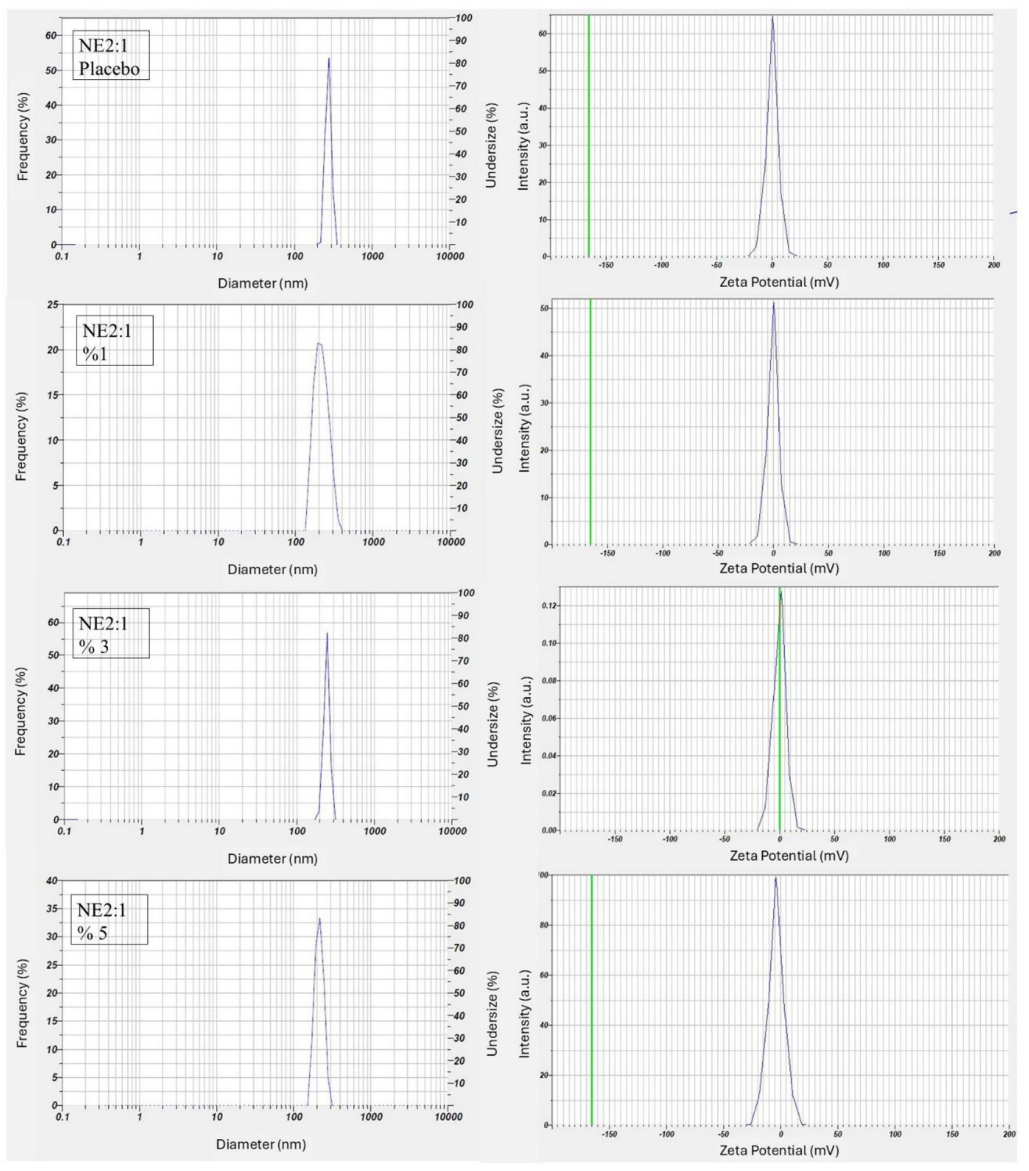
The droplet size distribution and zeta potential of nanoemulsion formulations.

All reported values correspond to the mean ± SD of three measurements. The average droplet size for 1%, 3%, and 5% NEs was 154.9 ± 9.6, 165.0 ± 3.6, and 151.3 ± 15.6 nm, respectively. The droplet size distribution and zeta potential graphs for each formulation are presented in Figure 2. The results of all formulations were within the nanometre range and exhibited the desired characteristics. In a previous study, various formulation combinations were designed using ethyl oleate, Cremophor EL, and Tween 80 to develop an NE containing corosolic acid. The distribution of droplet size and PDI of the prepared NE were characterized using DLS (Malvern Instruments, United Kingdom). The smallest droplet size of 181.3 nm and a PDI of 0.424 were obtained in formulations containing oleic acid (60).

The stability of NEs under storage is assessed by the zeta potential, which is an important parameter indicating the electrostatic surface charge of globules or particles. A higher absolute zeta potential value (both positive and negative) represents a higher stability. Stable NEs can also be obtained using non-ionic or polymeric surfactants, with zeta potentials as close to zero as possible (66, 67). The zeta potential measurement results showed that the formulations prepared with Cremophor EL, a non-ionic surfactant, did not exhibit any electrical charge (Table 3).

The results demonstrate that varying the concentration of the active ingredient within the NE 2:1 formulation has a limited impact on most physicochemical characteristics. Zeta potential values were close to zero for all samples, which may indicate steric stabilization provided by Cremophor^®^ EL rather than electrostatic repulsion. The stable pH and conductivity across all groups suggest that the internal structure of the nanoemulsions was not significantly altered by increasing active ingredient concentrations. Furthermore, viscosity remained within an acceptable range, indicating good flow properties suitable for topical or transdermal application. Overall, the data suggest that the NE 2:1 system is robust and tolerant to variations in active content within the tested range.

#### 3.3.2 Morphology of NEs

The surface morphology of the microemulsion formulations was investigated using a Leica light microscope (DM750). The sample was analyzed to determine the size of the NEs. The microscope images of the NEs revealed globular-shaped droplets with well-defined outlines. There was no aggregation. The sizes of the nanodroplets were consistent with the measurements obtained from the droplet size assessment, further supporting the accuracy of the size analysis (16).

#### 3.3.3 Value of pH

The pH values ranged from 5.0 to 6.0, as shown in Table 3. All formulations had a neutral pH value and did not exhibit highly acidic or highly basic characteristics.

#### 3.3.4 Conductivity

Conductivity is an important parameter to characterize emulsion type. The outcomes of the optimized formulations are presented in Table 3. The conductivity of w/o emulsions was usually low. This proved that the formulation was o/w type, confirming that the oil phase was indeed the inner phase and dispersed in the aqueous phase as desired (37).

#### 3.3.5 Rheological properties and viscosity

The main factors influencing emulsion viscosity are the colloidal interactions, droplet charge, droplet size, and volume fraction of the dispersed phase (26). The viscosity of the NEs incorporating EO at 1%, 3%, and 5% concentrations was 150, 250, and 350 mPa·s (millipascal-second), respectively, whereas the viscosity of the placebo formulation was 100 mPa·s. An ideal viscous emulsion was obtained compared with that in other studies on NEs (61, 62, 67).

**Figure 3:**
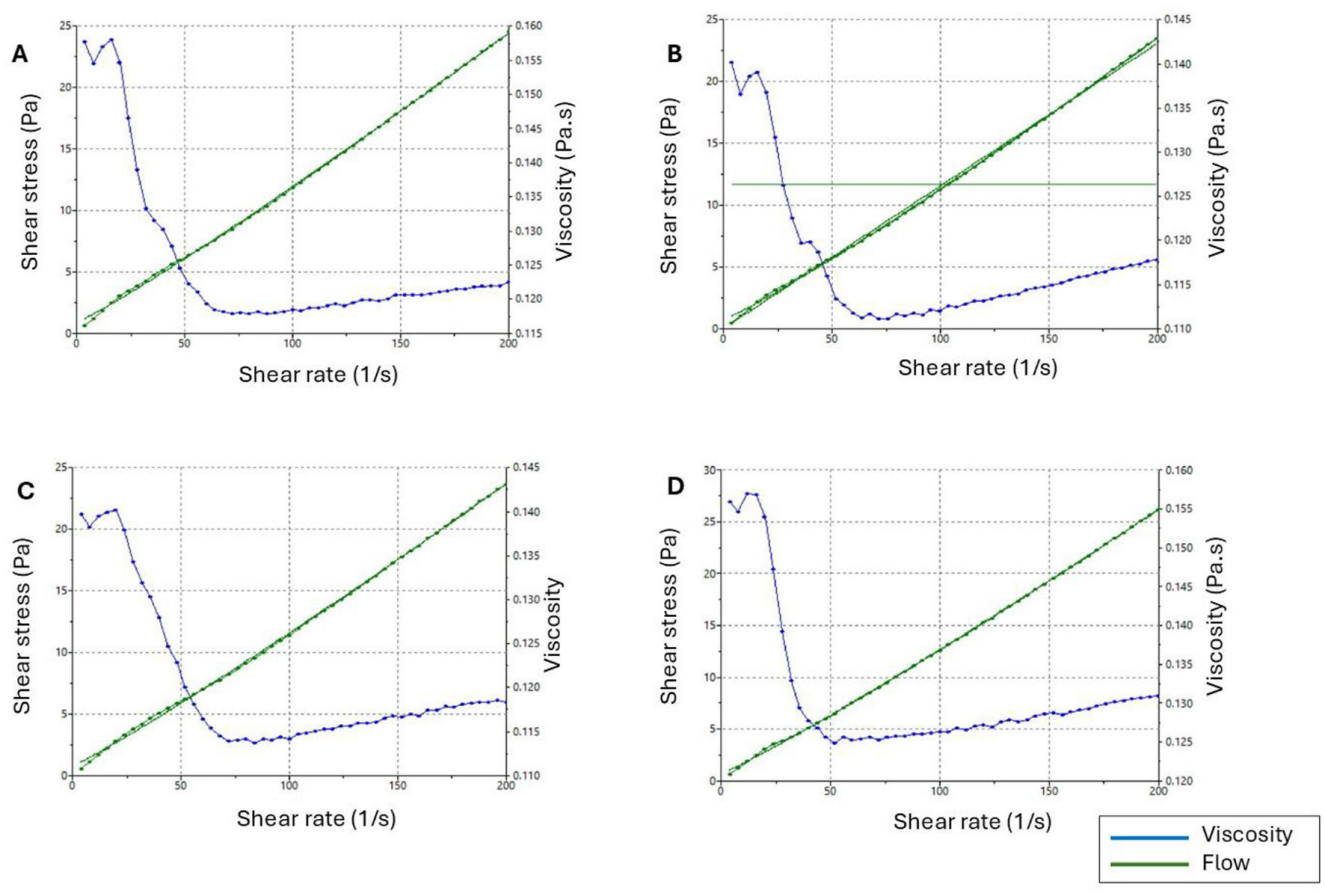
Rheograms showing the correlation between shear rate, viscosity, and shear stress. **(A)** NE2:1 Placebo, **(B)** NE2:1 1%, **(C)** NE2:1 3%, **(D)** NE2:1 5%).

The rheological behavior of the rheograms is shown in Figure 3. The shear rate increased proportionally with shear stress, but viscosity remained constant. This invariability confirmed that the NE behaved as a Newtonian fluid, suggesting a well-organized internal structure in which the components maintained their arrangement and resisted significant changes when subjected to applied stress. The linear increase in shear stress with shear rate, which is a characteristic of Newtonian fluids, confirmed the mechanical stability of the NEs. The steady rise in shear stress without deviation highlighted the robustness and consistency of the formulation under mechanical stress. The formulations were developed in accordance with the fluid nature of NEs, exhibiting low viscosity and aligning with the Newtonian fluid model (67–69).

### 3.4 Stability of NE formulations

The formulations can change (coalescence, cracking, creaming, precipitation, or phase separation) during long-term storage. Thus, stress tests, such as centrifugation and cycles of heating and cooling and freeze and thaw, were performed to explore the optimum formulations (37). Stable emulsions remained unchanged. The appearance and clarity were measured visually. In this study, the formulations maintained their original features when assessed against a blue background, indicating their long-term stability (61). In a comparable study, this was attributed to the order of the scale (nanometric), offering high kinetic stability, interfacial area, and optical transparency, and to the effect of Brownian motion, opposing gravity and favoring droplet separation (15).

Moreover, the other major benefit of using low-energy methods for preparing NEs is their spontaneous formation at room temperature, which does not need high energy, depending on the surfactant and cosurfactant mixture. It means no energy loss when conducting this reaction into the system, making it a less energy-consuming process. Usage is extremely important since NEs are influenced by both heat and high energy due to their thermodynamic stability. In particular, it must be emphasized that the stabilization of NEs is also ascribed to the adsorption of surfactant and cosurfactant molecules on the droplet surface, leading to repulsions between these structures via steric interactions (15, 70).

### 3.5 Antimicrobial activity analysis of EO and NE formulations

**Figure 4:**
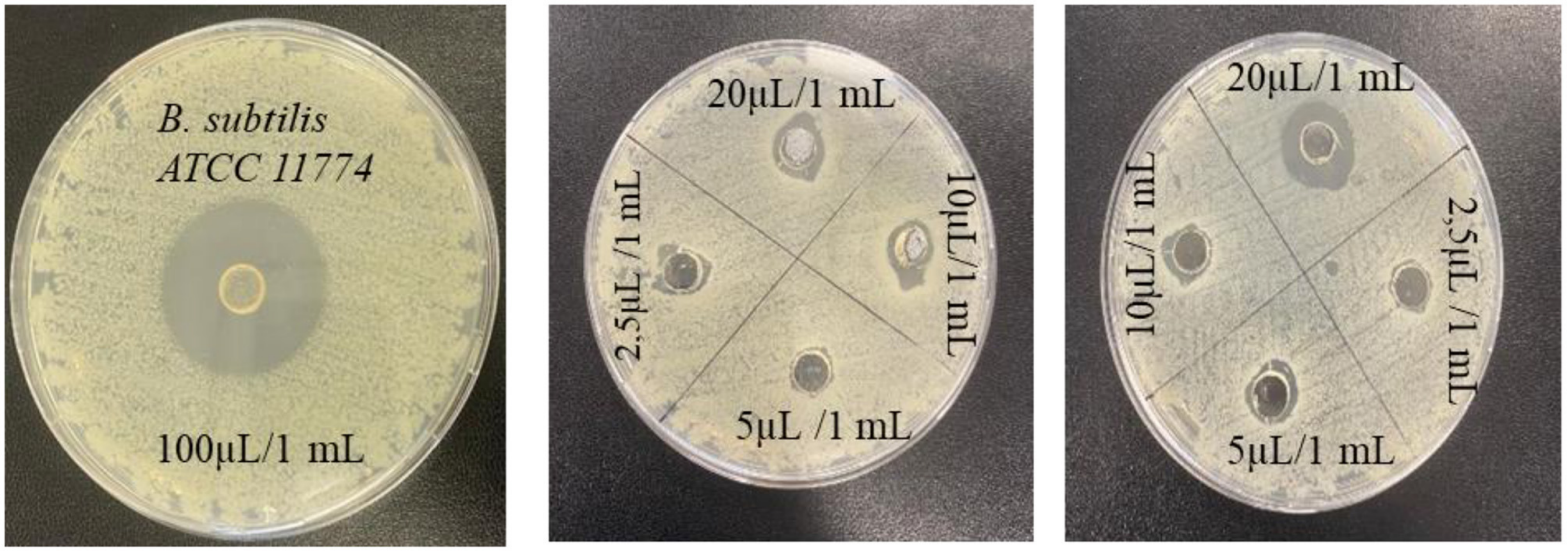
Antimicrobial activity of pure *Thymbra spicata* EO concentrations.

The antimicrobial activity of the EO of *T. Spicata* was tested against four different bacterial species using the agar well diffusion method (Table 4). On the tested microorganisms, the inhibition zone diameters of the EO varied in the range of 8.02 ± 41.18 mm, and it was found that it exhibited an inhibitory effect on all bacteria. In addition, the *T. spicata* EO showed high antimicrobial activity at concentrations ranging from even below 2.5 to 100 μl/ml. The highest inhibition zone was observed against *B. subtilis* at 41.28 mm, while the lowest inhibition effect was detected against *P. aeruginosa* at 11.22 mm. However, *E. coli ATCC 25922* formed an inhibition zone even at 0.3125 μl/ml concentration, while the other test bacteria (*B. subtilis ATCC 11774, P. aeruginosa ATCC 27853*, and *S. aureus ATCC 29213*) did not exhibit any antimicrobial activity at concentrations below 2.5 μl/ml. Moreover, 1%, 3%, and 5% *T. spicata* EO nanoemulsions did not show any antimicrobial activity against *S. aureus ATCC 29213, E. coli ATCC 25922, B. subtilis ATCC 11774* and *P. aeruginosa ATCC 27853* (Figure 4).

**Table 4:** Antimicrobial activity of various concentrations of pure *Thymbra spicata* EO.

**Concentration (μL/mL)**	**Inhibition zone diameter (mm)**			
	***E. coli***	***B. subtilis***	***S. aureus***	***P. aeruginosa***
100	33.99 ± 6.41	31.62 ± 2.88	29.78 ± 0.64	13.63 ± 1.78
20	15.78 ± 1.20	13.82 ± 1.34	14.18 ± 1.23	13.39 ± 1.67
10	13.65 ± 0.94	11.61 ± 1.21	12.41 ± 1.15	13.28 ± 1.97
5	11.53 ± 0.97	10.55 ± 0.5	9.93 ± 0.17	12.07 ± 2.23
2.5	10.91 ± 0.62	9.77 ± 0.36	9.64 ± 0.71	10.17 ± 0.54
1.25	11.69 ± 0.93	–	–	–
0.62	9.60 ± 0.5	–	–	–
0.31	8.34 ± 0.18	–	–	–

Kilic (71) reported that *T. spicata* EO exhibited antimicrobial activity against *B. subtilis, C. albicans, E. coli, E. faecalis, K. pneumoniae, P. aeruginosa, S. aureus, S. epidermidis*, and *S. typhimurium* microorganisms. The results of the study revealed that the EO has a high antimicrobial capacity against all tested microorganisms, with the exception of *P. aeruginosa*. The data obtained from this study support that the content of *T. spicata* has a broad range of antimicrobial activity, and they are in line with previous findings in the literature. In another study reported by Erturk et al. (72), *T. spicata* EO and extracts grown in Amasya produced zones of inhibition ranging from 10 to 43 mm against 10 different microorganisms, namely *A. niger, B. subtilis, C. albicans, K. pneumoniae, M. luteus, P. vulgaris, P. aeruginosa, S. aureus, Streptomyces murinus*, and *Y. enterocolitica*. Besides, in a study conducted by Sengun et al. (28), the antimicrobial activity, total phenolic content, and antioxidant capacity of *T. spicata* EO were higher than the plant extract. Thus, all test samples demonstrated antimicrobial activity against all bacteria at various levels, and the chemical composition of the EO showed that carvacrol was predominant. Karakaş and Bekler (73) examined *T. spicata* EO against *S. aureus ATCC 25923, E. coli ATCC 25922*, and *C. albicans ATCC 14053* with the disk diffusion method. A zone of inhibition of 36 ± 1.0 mm in diameter were found as the highest antimicrobial activities of *T. spicata* EO against *C. albicans*, a zone of inhibition of 26 ± 1.52 mm in diameter against *S. aureus* and a zone of inhibition of 28 ± 1.52 mm in diameter against *E. coli*. According to a study carried out by Kerem et al. (74), the antimicrobial activities of ethanol, hexane, and chloroform extracts of *T. spicata* were evaluated against *E. coli, E. faecalis, P. aeruginosa*, and *S. aureus* using the disc-diffusion method. The results showed that the ethanol and chloroform extracts of *T. spicata* were determined to be the most effective species against both the Gram-positive and Gram-negative strains (74). Barak et al. (25) developed a *T. spicata* EO using broth microdilution method partially exhibited against *S. aureus*.

While pure EOs exhibit immediate antimicrobial effects due to their direct interaction with microbial cells, encapsulating them into nanoemulsions can alter their release dynamics. Specifically, the encapsulation may lead to a controlled and sustained release of the active compounds, resulting in a delayed antimicrobial action compared to the rapid effect observed with pure EOs. This phenomenon has been reported in the literature. For instance, Donsì and Ferrari (75) discussed that nanoemulsions could provide a controlled release of EOs, which may prolong their antimicrobial activity over time but could also delay the onset of their effects. Similarly, Noori et al. observed that the antibacterial activity of geraniol nanoemulsions depended on the concentration and release rate of the EO from the emulsion, indicating that the encapsulation could modulate the timing and extent of antimicrobial action. Therefore, the lack of immediate antimicrobial activity in the NE form of the EO in our study could be attributed to the controlled release properties of the nanoemulsion, which may require a longer time to exhibit noticeable antimicrobial effects compared to the pure EO (76).

### 3.6 Application of the nanoemulsions on the curd cheese

The physicochemical properties of foods are very effective in determining their quality and how long they can be stored without spoiling (41).

#### 3.6.1 Physicochemical parameters of cheese samples during storage

Table 5 demonstrates how the physicochemical characteristics of curd cheese samples changed during 28 days of storage. The pH is one of the most important factors influencing the texture and flavor of cheese, as it affects the solubility of caseins and the activity of enzymes involved in ripening (39). The pH in all cheese samples varied between 6.54 ± 0.04 and 5.42 ± 0.01, indicating that nanoemulsions with *T. spicata* EO had a significant impact on the pH of curd cheese (*p* < 0.05). The release of alkaline compounds during proteolysis may explain the slight increase in the pH of the cheese, especially on day 5 (39). Additionally, in a recent study conducted to determine the effect of microencapsulated allspice nanoemulsion on the quality and shelf life of Ras cheese, total acidity increases and pH decreases in cheeses fortified with various concentrations of microcapsules emerging during the ripening period (77). Al-Obaidi (78) showed no significant differences in pH, ash, protein, fat, and moisture between normal cheese and cheese with three different amounts of *Curcuma longa* (0.1%, 0.2%, and 0.3%) immediately after production. Christaki et al. (79) determined the pH of oregano EO-based nanoemulsion at 4.28 and 6.34 in whey cheese. There were no significant differences in pH and other contents between the control and the trial, respectively, and curcumin nanoemulsion and emulsion-based cheese were also reported by Bagale et al. (80). Cai et al. (10) examined the pH of cheese increased in edible films reinforced with orange EO-based nanoemulsion during storage. The pH of cheese samples packaged with edible films increased slightly from 6.27 ± 0.014 on the first day to 6.465 ± 0.01 on the 12th day, indicating that no spoilage occurred in the cheese after 12 days of storage. The results showed that edible film packaging slowed down the pH changes of cheese, thereby preventing the cheese samples from spoiling. The particle size of nanoemulsions is closely related to WI. Hence, parameters such as droplet size, concentration, and refractive index directly affect the overall optical properties of the emulsion (81). In the study, all nanoemulsions visually exhibited a transparent appearance in cheese. However, WI measurements showed minor differences depending on the concentration of EO used in nanoemulsion (Table 5). In this study, the WI value decreased as the droplet size decreased. This situation reveals that the optical properties of nanoemulsions change depending on the particle size. The optical characteristics of nanoemulsions are an important factor when it comes to food applications (39). In most cases, transparent film-forming solutions are preferred to produce films that almost do not change the color of the food (82). Christaki et al. (79) incorporated nanoemulsions of oregano EO in whey cheese, and they observed that *C. citratus* or *C. reticulata* compared to *Origanum* or *Thymus* nanoemulsions (44.50 ± 0.17 and 32.94 ± 0.03), which had a significantly lower WI (27.89 ± 0.08 and 26.77 ± 0.49) (79).

**Table 5:** Effect of nanoemulsions on physicochemical characteristics of cheese samples during storage.

**Cheese samples**	**Storage days**					
	**3**	**5**	**7**	**14**	**21**	**28**
**pH**						
C	6.45 ± 0.02^Aa^	6.47 ± 0.01^Ba^	6.27 ± 0.02^BCb^	6.28 ± 0.01^Ab^	5.77 ± 0.17^Cc^	5.49 ± 0.01^Cd^
*T. spicata*	6.42 ± 0.01^ABb^	6.53 ± 0.02^Aa^	6.18 ± 0.01^Dc^	6.03 ± 0.02^Cd^	5.92 ± 0.01^Be^	5.62 ± 0.01^Bf^
Placebo	6.43 ± 0.03^ABb^	6.54 ± 0.04^Aa^	6.29 ± 0.01^Bc^	6.01 ± 0.02^Cd^	5.64 ± 0.02^Ce^	5.62 ± 0.02^Be^
%1	6.40 ± 0.02^Ba^	6.35 ± 0.03^Cb^	6.11 ± 0.02^Ed^	6.25 ± 0.04^Ac^	6.13 ± 0.02^Ad^	5.50 ± 0.01^Ce^
%3	6.34 ± 0.04^Ca^	6.33 ± 0.03^Ca^	6.35 ± 0.03^Aa^	6.20 ± 0.01^Bb^	5.92 ± 0.02^Bc^	5.42 ± 0.01^Dd^
%5	6.42 ± 0.03^ABb^	6.50 ± 0.01^ABa^	6.24 ± 0.04^Cc^	6.20 ± 0.02^Bd^	5.92 ± 0.01^Be^	5.79 ± 0.01^Af^
**WI**						
C	72.80 ± 0.01^Df^	88.33 ± 0.12^Aa^	79.78 ± 0.01^Bd^	81.43 ± 0.04^Bc^	84.19 ± 0.01^Ab^	78.79 ± 0.02^Be^
*T. spicata*	70.33 ± 0.01^Ef^	78.70 ± 0.03^Cd^	83.09 ± 0.03^Ba^	82.43 ± 0.02^Ab^	80.27 ± 0.02^Cc^	78.70 ± 0.04^Be^
Placebo	78.26 ± 0.17^Cc^	75.31 ± 0.01^Fe^	77.95 ± 0.04^Ed^	72.87 ± 0.08^Ff^	80.64 ± 0.01^Ba^	78.75 ± 0.07^Bb^
%1	69.57 ± 0.17^Ff^	76.68 ± 0.06^Ee^	83.12 ± 0.01^Aa^	77.54 ± 0.05^Ed^	79.43 ± 0.03^Ec^	82.50 ± 0.05^Ab^
%3	79.66 ± 0.07^Ab^	79.38 ± 0.02^Bc^	78.66 ± 0.01^Dd^	80.08 ± 0.07^Ca^	80.13 ± 0.04^Da^	76.76 ± 0.10^Ce^
%5	78.73 ± 0.01^Bc^	78.43 ± 0.04^Dd^	78.85 ± 0.04^Cb^	79.67 ± 0.05^Da^	77.07 ± 0.02^Fe^	74.91 ± 0.03^Df^
**Total phenolic content**						
C	16.39 ± 0.26^Ed^	14.25 ± 0.75^Dd^	20.28 ± 2.22^Ec^	41.84 ± 2.52^Eb^	60.167 ± 0.34^Da^	42.45 ± 1.55^Eb^
*T. spicata*	107.09 ± 0.09^Ae^	116.33 ± 10.12^Ad^	165.08 ± 1.25^Aa^	134.50 ± 3.66^Ac^	164.75 ± 1.75^Aa^	147.25 ± 6.25^Ab^
Placebo	28.67 ± 3.75^Dc^	19.39 ± 3.87^CDc^	28.89 ± 4.03^Dc^	56.44 ± 4.98^Db^	63.22 ± 9.20^CDb^	73.72 ± 4.62^Da^
%1	31.84 ± 6.21^De^	27.00 ± 1.67^Ce^	45.17 ± 1.67^Cd^	53.17 ± 3.08^Dc^	67.94 ± 2.80^BCDb^	88.72 ± 1.25^Ca^
%3	37.94 ± 2.12^Cd^	45.28 ± 4.67^Bc^	19.05 ± 1.55^Ee^	73.167 ± 0.34^Cb^	72.00 ± 4.10^BCb^	84.42 ± 2.09^Ca^
%5	97.28 ± 0.19^Ba^	50.11 ± 5.01^Bd^	64.22 ± 2.64^Bc^	80.33 ± 3.06^Bb^	72.78 ± 5.91^Bb^	105.17 ± 7.37^Ba^
**ABTS**						
C	293.87 ± 0.65^Da^	157.78 ± 3.14^Eb^	149.96 ± 8.82^Ebc^	133.99 ± 9.25^Ec^	136.13 ± 16.46^Dc^	48.27 ± 9.90^Ed^
*T. spicata*	897.93 ± 0.75^Aa^	861.60 ± 26.22^Aa^	864.36 ± 0.62^Aa^	536.00 ± 37.00^Ac^	745.7 ± 28.10^Ab^	671.00 ± 32.80^Ad^
Placebo	314.73 ± 0.06^Da^	145.29 ± 2.62^Ec^	135.50 ± 23.67^Ec^	267.56 ± 18.50^Cb^	134.53 ± 8.54^Dc^	32.89 ± 5.05^Ed^
%1	337.60 ± 1.10^Da^	224.98 ± 3.39^Db^	202.44 ± 1.87^Db^	193.12 ± 9.53^Db^	180.89 ± 5.94^Db^	89.49 ± 13.49^Dc^
%3	532.18 ± 59.30^Ca^	336.36 ± 3.54^Cb^	267.56 ± 10.84^Cbc^	266.49 ± 9.88^Cb^	263.60 ± 8.24^Cbc^	192.00 ± 22.00^Cc^
%5	634.6 ± 74.45^Ba^	512.53 ± 15.24^Bb^	426.98 ± 6.17^Bc^	335.60 ± 38.88^Bde^	364.84 ± 15.05^Bcd^	283.80 ± 13.80^Be^

Results are presented as mean ± SD. Significant differences exist between the averages in a row with different lowercase letter superscripts (*p* < 0.05) (a–d). Significant differences exist between the averages column, with differences in each upper case superscripted (*p* < 0.05).

C, Control.

#### 3.6.2 Antioxidant activity and total phenolic content analysis

TPC and the antioxidant activity of curd cheese fortified with *T. spicata* EO nanoemulsion during the stored period were given in Table 5. In this study, the antioxidant capacity (15.47 ± 1.05), which was very low on the first day in the control sample, increased on the third day and started to decrease on the following storage days (*p* < 0.005). *T. spicata* pure oil has the highest antioxidant activity starting from the third day (Table 5). However, an increasing trend was obtained in antioxidant activity of 1%−5% EO-added nanoemulsion (*p* < 0.005). The results obtained from this study are in agreement with the literature. The study conducted on the antioxidant activities of Kareish cheese that was supplemented with curcumin nanoemulsions at 1.0, 1.5, and 2.0 mg/g showed the highest antioxidant activity in the nanoemulsion at 2.0 mg/g. Shawir et al. reported that the antioxidant activity of kareish cheese was between 19.23 and 42.31% and the antioxidant effects of kareish cheese applied with curcumin nanoemulsions at 1.0, 1.5, and 2.0 mg/g were 33.40%, 37.80%, and 42.31%, respectively (41).

Although all antioxidant activities decreased over time during storage, significant activity was detected in *T. spicata oil* and 1%, 3%, and 5% nanoemulsions on the 28th day of storage than the control cheese sample. In addition, the change in antioxidant capacity results was correlated with the total phenolic content of nanoemulsions (Table 5). Moreover, in a study, El-Sayed et al. (83) aimed to increase the phenolic compound and antioxidant content in Ras cheese by the addition of microencapsules containing nanoemulsions of allspice fruit extracts. The results revealed that the control cheese had lower TPC content (6.26 mg/100 g) and antioxidant activity (9.89%) than the supplemented cheese samples. TPC and antioxidant activity increased significantly (*p* < 0.05) with increasing microcapsule concentrations in Ras cheese. The total antioxidant activity, total phenolic content, and radical scavenging activity of *T. spicata* were analyzed by Bener (84). All models calculated for the three responses that are total phenolic, antioxidant, and radical scavenging activities were noteworthy (*p* < 0.0001) and showed that there was a significant relationship between the response and the independent parameters (84). The antioxidant capacity of *T. spicata* EO-based nanoemulsions on cheese has not been studied, but it has been studied on cheese with nanoemulsions containing EO from different plant species. Pérez-Soto et al. incorporated phenolic compounds and the antioxidant capacity of microemulsions from *Opuntia oligacantha* to fresh cheese, and they observed that phenolic compounds decreased during storage. As observed with ABTS, the microemulsion maintained a higher antioxidant acitivity with DPPH from day 0 (19.50 ± 0.43mg AAE/g). This behavior was observed up to 45 days (85). Shabani et al. (86) investigated the antioxidant activities of *Urtica dioica* EO-based nanoemulsions on *L. monocytogenes* and *E. coli* and reported that the values of DPPH were identified in EO-based nanoemulsions as 33.7 ± 12.2. Faramarzi et al. investigated a similar study with Shabani et al. (86) and applied it to the same bacteria, and the effect of *Urtica dioica* EO and nanoemulsions was identified in the nanoemulsions as 31.25 ± 1.50 % (11).

#### 3.6.3 Microbiological analysis of cheese samples during storage

**Figure 5:**
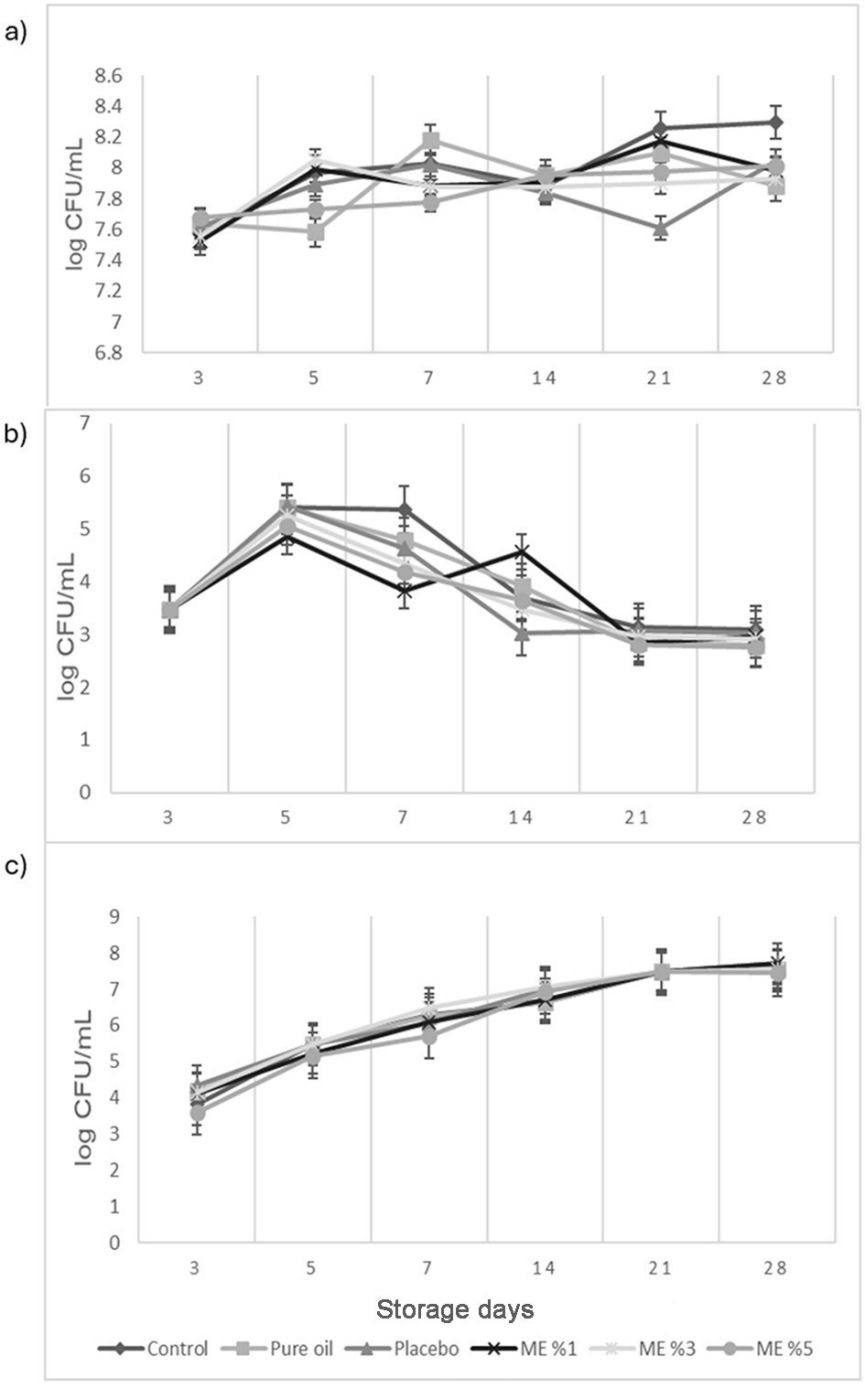
**(a)** Total mesophilic aerobic bacteria; **(b)** Lactic acid bacteria; **(c)** Yeast counts of cheeses during storage.

Microbiological analyses of curd cheeses were performed on each storage day. Total mesophilic aerobic bacteria (TMAB) counts, lactic acid bacteria (LAB), and yeast counts of curd cheeses during storage were given in Figures 5a–c, respectively. TMAB counts were found between 7.58 and 8.18 log cfu/g in curd cheese with pure oil added, 6.97 and 8.29 log cfu/g in control curd cheese sample, 7.64 and 8.04 log cfu/g in placebo added curd cheese, and 7.52 and 8.17, 7.55 and 8.05, and 7.68 and 8.01 log cfu/g in curd cheese sample containing 1%, 3%, and 5% *T. spicata* EO nanoemulsion, respectively. The total number of aerobic mesophilic bacteria in the curd cheese to which different EOs (1%, 3%, and 5%) containing nanoemulsions were added before storage decreased slightly. LAB counts were found between 2.80 and 5.41 log cfu/g in curd cheese with pure oil added, 2.21 and 5.41 log cfu/g in control curd cheese sample, 3.02 and 5.42 log cfu/g in placebo added curd cheese, and 2.84 and 4.85, 2.91 and 5.26, and 2.81 and 5.05 log cfu/g with 1%, 3%, and 5% *T. spicata* EO nanoemulsion, respectively. Yeast counts were found between 4.17 and 7.54 log cfu/g in pure oil-added curd cheese, 3.82 and 7.49 log cfu/g in control curd cheese sample, 4.35 and 7.72 log cfu/g in placebo-added curd cheese, and 4.13 and 7.70, 4.13 and 7.54, and 3.59 and 7.44 log cfu/g in 1%, 3%, and 5% *T. spicata* EO nanoemulsions, respectively. It was observed that the number of yeasts increased as the storage was prolonged. No mold growth was observed in the curd cheese sample. El-Sayed et al. reported the preservation effect of cumin EO nanoemulsion as a brined solution on the microbiological properties of white soft cheese during 60 days of storage time (77). The total mesophilic bacterial counts and lactic acid bacteria counts of white soft cheese treatments gradually reduced significantly in the presence of cumin EO nanoemulsion. However, it is observed that this decrease was more noticeable after a 30-day storage period. The reason for the small change in this study could be related to the shorter period of storage time for cheese samples. Elsherif and Al Shrief studied the effectivity of *Szygium aromaticum* and *Cuminum cyminum* EOs and its nanoemulsions against foodborne pathogens after inoculation in manufactured cheese at different concentrations. Complete reduction of *L. monocytogenes* was observed after 2 weeks of treatment with carvacrol nanoemulsions at 0.78 and 1.56% (2).

Gadallah et al. showed antimicrobial and antioxidant properties of *Rosmarinus officinalis* EO-based nanoemulsions as a natural alternative to Karish cheese. The results demonstrated a complete reduction of *A. flavus, B. cereus*, and *L. monocytogenes*, on days 5, 7, and 10, respectively, and a 96.93% reduction of *P. aeruginosa* at the end of the storage time. In the viability study of Karish cheese, *C. albicans, A. flavus*, and *P. aeruginosa* were completely reduced on days 10, 10, and 15 of storage, respectively (4).

Abd El Gwad et al. developed *Thymus vulgaris* and *Syzygium aromaticum* EOs-based nanoemulsions to improve the quality of soft cheese. The results showed that different concentrations of *T. vulgaris* nanoemulsion (0.03%, 0.06%, and 0.1%), as well as *S. aromaticum* nanoemulsions (0.06%, 0.1%, and 0.25%), were used to preserve the cheese. During a cold storage period of 50 days, the microbiological analysis and sensory evaluation of different cheeses were evaluated. The results showed that the droplet size of the prepared NEs was 94.85 and 68.67 nm for *T.vulgaris* and *S. aromaticum* nanoemulsions, respectively (3). Kamal et al. investigated the inhibitory effect of thyme oil against *C. sakazakii* in Tallaga cheese. Furthermore, thyme oil also inhibited the growth of *Salmonella* and mesophilic aerobic bacteria in other food products, maintained until the end of storage at 4°C (87). Shawir et al. showed that increasing the concentration of *Curcumin* nanoemulsions (1.0, 1.5, and 2 mg/ml) can be used in the preservation of Kareish cheese to enhance antimicrobial protection (41).

## 4 Conclusion

*T. spicata* EOs showed antimicrobial activity against all bacteria tested (*Bacillus subtilis, Escherichia coli, Pseudomonas aeruginosa*, and *Staphylococcus aureus*). The strong antibacterial properties of *T. spicata* EO may be due to its thymol and carvacrol components. Despite its low stability, the nanoemulsion demonstrated a strong antioxidant activity on the cheese. Of particular importance, the antibacterial activity of the EO decreased against tested bacterial strains when it was converted to a nanoemulsion. The nanoemulsion concentrations of 1%, 3%, and 5%, as well as pure EO were incorporated into curd cheese; the nanoemulsion delayed mold and yeast growth up to 28 days.

The chemical profile identified in this study suggested that *T. spicata* EO could serve as a natural preservative or antimicrobial agent in the pharmaceutical and food sectors. The high content of bioactive monoterpenes supports the potential use of *T. spicata* EO in therapeutic formulations targeting microbial infections and oxidative stress.

Future studies should explore the seasonal variability in the EO composition and its effect on biological activities. Additionally, comparative analyses with EOs from different ecological zones may provide further insight into the impact of environmental factors on the chemical profile of *T. spicata*.

## Data Availability

The raw data supporting the conclusions of this article will be made available by the authors, without undue reservation.
